# Phytochemical-Rich Germinated Oats as a Novel Functional Food To Attenuate Gut Inflammation

**DOI:** 10.1021/acs.jafc.5c02993

**Published:** 2025-06-12

**Authors:** Pei-Sheng Lee, Juanjuan Hu, Shengmin Sang

**Affiliations:** 1 Laboratory for Functional Foods and Human Health, Center for Excellence in Post-Harvest Technologies, North Carolina Agricultural and Technical State University, Kannapolis, North Carolina 28081, United States; 2 Center for Gastrointestinal Biology and Disease, University of North Carolina, Chapel Hill, North Carolina 27599, United States

**Keywords:** oat germination, avenanthramides, avenacosides, avenacins, gut inflammation

## Abstract

Oat (Avena sativa L.) is rich in phytochemicals such as avenanthramides, avenacosides, and avenacins, which support intestinal health and exhibit antioxidative and anticancer properties. Germination enhances these phytochemicals, potentially increasing their efficacy. To evaluate the anti-inflammatory activity of germinated oats, various germinated oat products were screened for anti-inflammatory activity using an LPS-induced nitric oxide assay in RAW 264.7 macrophages. The most effective sample was further tested in a dextran sulfate sodium-induced colitis mouse model. Results showed that germinated oat extract significantly reduced inflammation-related symptoms and cytokines (IL-6, TNF-α, IL-1β, TGF-β, and cyclooxygenase-2) compared to those of raw oats. LC/MS analysis confirmed elevated levels of oat phytochemicals in both germinated oats and the feces of mice treated with germinated oats. Germination significantly increased the concentrations of major bioactive oat phytochemicals, and mice consuming germinated oats had higher levels of these compounds. Furthermore, correlation analysis revealed a strong negative association between inflammation markers and phytochemicals, especially avenanthramides and their metabolites. These findings suggest that germination enhances the phytochemical content of oats, thereby enhancing their anti-inflammatory abilities in both cell and animal models of colitis, indicating that germinated oats could serve as a value-added functional food for reducing gut inflammation.

## Graphical abstract

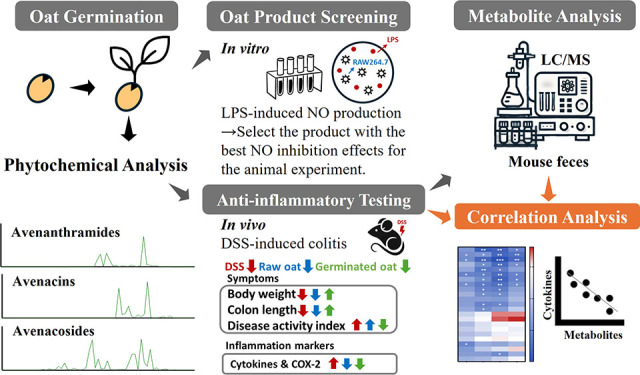

## Introduction

Oats have been cultivated worldwide for over 2000 years and are recognized as versatile crops with a superior nutritional value compared to many other cereals. The U.S. FDA claims that oats, as part of an overall heart-healthy diet, can reduce the risk of heart disease. Numerous laboratory and clinical studies have indicated that oat consumption effectively lowers serum cholesterol levels, reduces glucose uptake, and attenuates plasma insulin response.^1–3^

Oats are typically consumed as whole grains, providing essential nutrients such as proteins, unsaturated fatty acids, vitamins, minerals, and β-glucan.^4^ Among these nutrients, β-glucan is a key active component known for its cholesterol-lowering and antidiabetic properties.^5,6^ However, the health benefits of oats extend beyond their fiber content. Oats are also rich in various bioactive phytochemicals, which are secondary metabolites synthesized by plants and exhibit diverse structures. Similar to other grains, oats are a rich source of phenolic acids, which exhibit strong antioxidant and anti-inflammatory properties, contributing to the protection against chronic diseases and supporting overall health.^7^ Additionally, oats produce three unique types of phytochemicals: avenanthramides (AVAs), avenacosides (AVEs), and avenacins (AVCs).^8,9^ AVAs are phenolic compounds containing substituted *N*-cinnamoylanthranilic acids, where 2c, 2p, and 2f are the most abundant (C-type AVAs) and *N*-avenalumoylanthranilic acids, where 2cd, 2pd, and 2fd are predominant (A-type AVAs) (Figure 1).^8,10^ AVAs have been reported to exhibit multiple bioactivities, including anti-inflammatory effects,^10,11^ cancer prevention,^12^ cardiovascular disease risk reduction,^13,14^ allergic disease mitigation,^15–17^ gut microbiota modulation,^18–20^ and attenuation of metabolic syndrome.^21^

**Figure 1:**
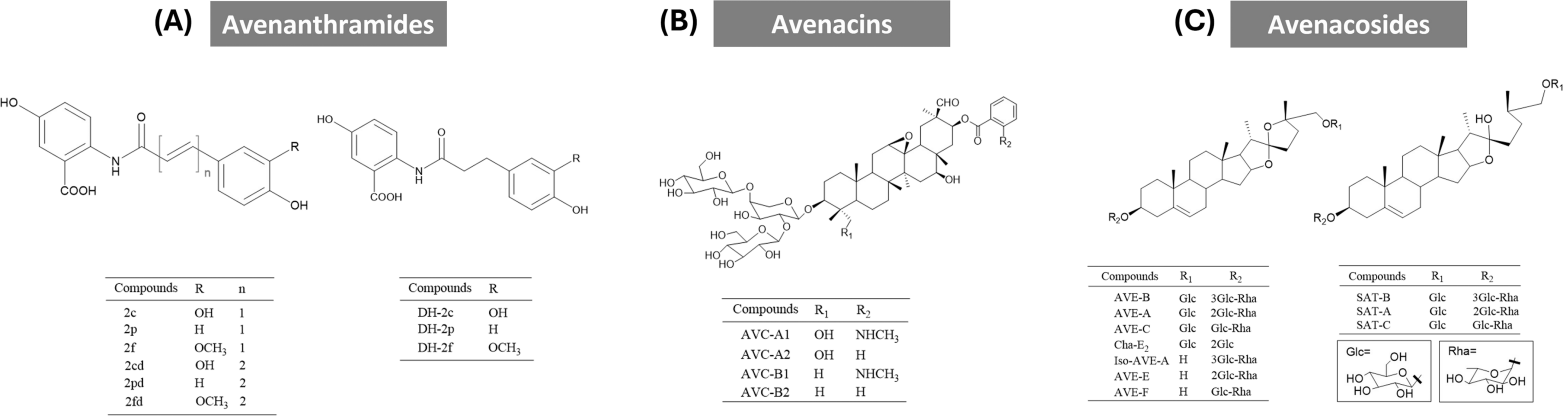
Chemical structures of avenanthramides (A), avenacins (B), and avenacosides (C) in oats.

AVEs and AVCs are steroidal saponins and triterpenoid saponins, respectively. AVE-A and AVE-B are the major AVEs, while AVC-A1, AVE-A2, AVE-B1, and AVE-B2 are the primary triterpenoid saponins found in oats (Figure 1). Due to the lack of commercially available standards, research on the health benefits of AVEs remains limited, and no studies have been conducted on the potential health effects of AVCs. Our recent *in vitro* study reported AVE-E (Figure 1), the deglycosylated product of AVE-A and AVE-B, shows the most potent anti-inflammatory activity among all major and minor AVEs by inhibiting nitric oxide (NO) production in LPS-treated RAW264.7 cells. Furthermore, germination is a cost-effective and natural processing method that activates enzymatic and metabolic processes in grains, leading to the breakdown of macronutrients and the synthesis of bioactive compounds. In oats, germination has been shown to enhance the levels of beneficial phytochemicals, such as AVAs and phenolic acids,^22,23^ thereby improving their antioxidant, anti-inflammatory, and gut health-promoting properties. These advantages support the development of germinated oat-based functional foods with enhanced health benefits.^20^

This study aims to test our hypothesis that germinated oats exert stronger anti-inflammatory effects than raw oats due to their higher levels of bioactive phytochemicals. First, the NO production assay was used to screen commercially available oat seed products and identify the product with the highest anti-inflammatory activity after germination. The selected oat seed product was then produced in larger quantities and further evaluated in an *in vivo* study using the dextran sulfate sodium (DSS)-induced colitis mouse model to compare the anti-inflammatory effects of phytochemical extracts from germinated and raw oats.

## Materials and Methods

### Chemicals and Reagents

All 22 oat seed products were obtained from online vendors (Table S1). The 22 commercial oat seed products (Brands 1-22) included in this study were selected to represent a broad diversity of growing regions (across various U.S. states), planting seasons, species (primarily Avena sativa L. and Avena nuda), and intended uses (e.g., forage, planting, and human consumption). Our goal was to capture the real-world variability found in commercially available oat seeds that are accessible to consumers and producers, particularly those marketed for planting or dietary applications. Rather than a controlled breeding study, our selection reflects the heterogeneity of oat products on the market.

Reversed-phase C18 columns (Biotage Sfär C18 D, 240 g, Duo 100 Ǻ 30 μm) and an SPE column (GX-274 ASPEC, Gilson, Middleton, WI, USA) were used for open column chromatography. All analytical-grade solvents and liquid chromatography–mass spectrometry (LC–MS) grade solvents were obtained from Thermo Fisher Scientific (Waltham, MA, USA.). All oat authentic standards (AVAs and AVEs) were previously purified or synthesized in our lab with a purity greater than 95%.^9,10,24–27^ Tranilast (Sigma-Aldrich, St. Louis, MO, USA) and glycyrrhizic acid (Sigma-Aldrich, St. Louis, MO, USA) were used as the internal standards for AVAs and AVEs/AVCs, respectively.^9,11^ The cyclooxygenase-2 (COX-2) antibody (Catalog number: 160112) was procured from Cayman Chemical (Ann Arbor, MI, USA). The mouse β-actin monoclonal antibody (catalog number: 4970S) was purchased from Cell Signaling Technology (Danvers, MA, USA). The lipopolysaccharides (LPS, derived from *Escherichia coli* O111:B4, Catalog number: L4391) were obtained from Sigma Chemical Co. (St. Louis, MO, USA).

### Preparation of Germinated Oats

Raw oat seeds (7 g each) from 22 different oat seed products were processed in full accordance with the germination method described by Hu et al.^28^ The seeds were first disinfected with a 1.5% sodium hypochlorite solution, thoroughly rinsed, and then evenly spread on 15 cm Petri dishes, which were covered and placed in a germination chamber maintained at 60% relative humidity in darkness. Sampling was conducted on the fifth day at 20 °C, as previous experiments indicated that these conditions yielded the highest total content of AVAs, AVEs, and AVCs. After germination, the seeds were dried in an oven at 60 °C and subsequently ground into a powder for later use.

### Preparation of Phytochemical-Rich Extracts from Raw and Germinated Oats

For *in vitro* cell experiments, powdered raw or germinated oats (1 g per sample) were accurately weighed and extracted with 50% ethanol (12.5 mL) in water for 12 h, with the process repeated four times to obtain approximately 45 mL of extract. At least three independent samples of each oat seed product were used in this study. From each replicate, 20 mL of each extract was picked to yield a combined total of 60 mL per sample. The combined extract was then concentrated into a crude extract. The concentrated extract was loaded onto a 30 mg SPE column and sequentially washed with 30%, 50%, and 100% methanol. The 100% methanol fraction, found to be rich in AVAs, AVEs, and AVCs via LC-MS analysis, was collected, further concentrated, and dried into a solid form for use in cell anti-inflammatory experiments.^28^

For *in vivo* mouse experiments, 200 g of Brand 2 oat seeds were germinated on a tray using the aforementioned procedure, yielding 176 g of germinated oats after drying. Both the raw and germinated oats were ground into powder and soaked in 50% ethanol (*V*_oats_:*V*_50% EtOH_ = 1:5) for 12 h, with the process repeated four times. The combined extracts were then concentrated and purified using a C18 column (240 g packing) to accommodate the large sample load. The column was sequentially eluted with 30, 50, 80, and 100% methanol. The 80% methanol fraction, which was rich in target compounds via LC-MS analysis, was concentrated and used as a feed material for the *in vivo* mouse experiments.

### LC–MS/MS Analysis

Chemical quantification: The quantification of AVAs, AVCs, and AVEs in all oat extracts and fecal samples was performed following the LC–MS/MS method adapted from Hu et al.^28^ The analysis was conducted on a Thermo-Finnigan Spectra System coupled to an LTQ Velos Pro ion trap mass spectrometer incorporating an electrospray ionization (ESI) source. Chromatographic and mass spectrometric conditions were maintained as described in the original method, utilizing a Gemini C18 instrument (150 × 3.0 mm i.d., 5 μm). Mass spectrometric parameters were optimized using standard solutions of AVE-E, SAT-C, 2c, and 2f (500 nM in methanol) with tuning adjustments made to achieve optimal sensitivity and accuracy.

Chemical profiles: To further characterize the 30%, 50%, and 100% methanol (MeOH) fractions of raw and germinated oat samples following SPE purification, chromatographic separation was optimized using a Gemini C18 (50 mm × 2 mm i.d., 3 μm) column from Phenomenex (Torrance, CA, USA). The gradient elution was applied as follows: 0–1 min, 15% B; 1–8 min, 15–45% B; 8–13 min, 45–100% B. Re-equilibration was performed with 15% B from 14.1 to 15 min. All of the other parameters remained consistent with those used for sample quantification. All mass spectrometric data were processed using an Xcalibur 4.0 (Thermo Electron, San Jose, CA, USA).

### Cell Culture Conditions and Treatments

RAW264.7 (ATCC TIB-71) cells were obtained from the American Type Culture Collection (Rockville, MD, USA). RAW264.7 cells were grown in ATCC-formulated Dulbecco’s modified Eagle’s medium (DMEM) containing 10% endotoxin-free, heat-inactivated fetal bovine serum (FBS). The cells were cultured in a serum-free medium for the inflammation experiments. Oat extracts (40 μg/mL) dissolved in dimethyl sulfoxide (DMSO) and lipopolysaccharide (LPS, 100 ng/mL) were added. The nitrite concentration was measured as an indicator of NO production, according to the Griess reaction. The absorbance of the mixture at 545 nm was measured by using an ELISA reader. The results were normalized to those of the corresponding control. The oat extracts were cotreated with LPS.

### Animal Experimental Design

Six-week-old male C57BL/6J mice were purchased from the Jackson Laboratory and housed in a controlled environment at 20 ± 2 °C with relative humidity of 50 ± 10%, and a 12 h light–dark cycle. The animal protocol used in this study was approved by the Institutional Animal Care and Use Committee of the North Carolina Research Campus (Protocol #20–007).

The mice were randomly assigned to five groups: a normal diet (ND) control group, a colitis model group receiving a normal diet with 2.5% dextran sulfate sodium (DSS), a DSS-treated group supplemented with 0.2% phytochemical extract from raw oats, which is equivalent to 21% raw oats in the diet based on the percentage of the phytochemical extract obtained from raw oats (DSS + OAT), a DSS-treated group supplemented with 0.1% phytochemical extract from germinated oats, which is equivalent to 7% germinated oats in the diet based on the percentage of the phytochemical extract obtained from germinated oats (DSS + LG-OAT), and a DSS-treated group supplemented with 0.3% phytochemical extract from germinated oats, which is equivalent to 21% germinated oats in the diet (DSS + HG-OAT).

The sample dose selection was based on recommendations from the 2020–2025 Dietary Guidelines for Americans (https://www.dietaryguidelines.gov/resources/2020-2025-dietary-guidelines-online-materials). The guideline states that for a healthy U.S.-style dietary pattern at a 2000 calorie level, a daily intake of 6 ounces of grains is recommended, with at least 3 ounces (84 g) coming from whole grains (WGs). For a 60 kg human, consuming 3 ounces of WGs per day translates to a 17.2 g/kg daily dose in mice.^29^ Given that the daily food intake of a 20 g mouse is approximately 2.5 g, the 17.2 g/kg daily dose corresponds to 14% of the total diet as WGs. Therefore, the 7 and 21% WG equivalent doses used in this study are relevant to human consumption. Since the extraction yield of phytochemicals from raw oats is lower than that from germinated oats, the 21% whole-oat equivalent dose corresponds to 0.2% phytochemical-rich extract for raw oats and 0.3% for germinated oats.

Colitis was induced by administering 2.5% DSS (molecular weight 36–50 kDa, MP Biomedicals, LLC) in drinking water for 5 days, followed by 7 days of recovery with regular water. Mice in the treatment groups received phytochemical-rich extracts from either raw or germinated oats for 2 weeks prior to DSS exposure. The experimental design is summarized in Figure 5A.

Mice had ad libitum access to food and water throughout the study. At the end of the experiment, they were anesthetized, and their blood was collected through cardiopuncture. Feces from the colon of each individual mouse as well as colon tissues were immediately harvested, weighed, and frozen for further analysis.

### Disease Activity Index and Cytokine Analysis

The disease activity index (DAI) scores were used to assess the severity of the colitis. These scores included (i) body weight loss, (ii) stool consistency, and (iii) hematochezia. Each score was determined as follows: change in body weight loss (0: none, 1:1–5%, 2:5–10%, 3:10–15%, 4: >15%); stool consistency (0: normal, 1: moist or sticky stool, 2: soft stool, 3: soft stool with mild diarrhea, 4: diarrhea); and hematochezia (0: negative, 1: Hemoccult-positive, 2: Hemoccult-positive with visual pellet, 3; moderate blood, 4: gross bleeding). Body weight loss was calculated as the percent difference between the original body weight (day 0) and the body weight on any particular day. The detection of occult blood was performed using the Hemoccult guaiac fecal occult blood test kit (Beckman Coulter) according to the manufacturer’s instructions.

Cytokine levels were measured using the relevant ELISA kits, following the manufacturer’s instructions. Plasma was analyzed for IL-6 (OptEIA mouse IL-6 ELISA kit, Becton Dickinson), TGF-β (mouse TGF beta 1 ELISA kit, Invitrogen), IL-1β (mouse IL-1 beta Quantikine ELISA kit, R&D Systems), and TNF-α (mouse TNF-alpha Quantikine ELISA kit, R&D Systems).

### Western Blot Analysis

For protein analysis, the total colon was homogenized on ice with a bead mill homogenizer (Omni International, Kennesaw, GA) and lysed with ice-cold lysis buffer (Cell Signaling Technology). The mixture was then centrifuged at 16500 × *g* for 30 min at 4 °C. Protein content was measured using a Pierce BCA assay kit (Thermo Fisher Scientific). The total protein (25 μg) was subjected to SDS-polyacrylamide gel electrophoresis and transferred to polyvinylidene fluoride (PVDF) membranes. The membranes were blocked for 1 h at room temperature with 5% milk (Bio-Rad Laboratories, Berkeley, CA) and incubated with the COX-2 primary antibodies (BD Transduction Laboratories) overnight. Blots were then washed with TBS-Tween 20 and probed for 1 h with the appropriate horseradish peroxidase (HRP)-conjugated secondary antibody (1:5000). Protein bands were visualized via chemiluminescence using a West Femto maximum detection substrate (Thermo Fisher Scientific). To confirm equal protein loading in each lane, β-actin (Cell Signaling Technology) was used as the loading control. Protein fold-induction was calculated by normalizing the intensity of the band of interest to β-actin first and then comparing it to the control lanes by using ImageJ imaging software (National Institutes of Health).

### Fecal Sample Preparation

Mouse feces were dried and ground into powder. Fifteen mg of fecal powders were soaked in 300 μL of 90% MeOH containing 0.1% acetic acid and subsequently homogenized for 9 min by the bead mill homogenizer. The resulting suspension was centrifuged at 16100 × g for 20 min, and 100 μL of the supernatant was spiked with 100 μL of internal standards (tranilast and glycyrrhizic acid) and 100 μL of MeOH. The final mixture was directly injected into the LC-MS for analysis. Each sample was analyzed in duplicate.

### Quantification of AVAs, AVEs, and AVCs in Oat Extracts and Mouse Fecal Samples

Individual stock solutions of the nine AVAs (2c, 2p, 2f, 2cd, 2pd, 2fd, DH-2c, DH-2p, and DH-2f) and eight AVEs (AVE-A, AVE-B, AVE-C, AVE-E, AVE-F, SAT-A, SAT-B, and SAT-C) were prepared in methanol at 10 mM. These external standard solutions were then combined and serially diluted with methanol to create a mixed standard solution with analyte concentrations ranging from 0.0012 to 50.0 μM. Additionally, two internal standard stock solutions (tranilast for AVAs and glycyrrhizic acid for AVEs/AVCs) were prepared in methanol at 10 mM. These solutions were diluted and combined into a single working solution, with each at a final concentration of 100 and 300 nM, where 100 nM was used for oat extract quantification and 300 nM for fecal sample quantification. Due to differences in the sample matrices, separate calibration curves were established for oat extracts and mouse fecal samples.

For oat extracts, the calibration curve was constructed by mixing 100 μL of the external standard mixture with 100 μL of the internal standard solution. Sample preparation involved combining 100 μL of the oat extract with 100 μL of the internal standard solution before analysis.

For mouse fecal samples, calibration standards were prepared by mixing 100 μL of a fecal extract from the ND group with 100 μL of the external standard mixture and 100 μL of the internal standard solution. Similarly, for sample analysis, 100 μL of fecal extract was mixed with 100 μL of methanol and 100 μL of the internal standard solution prior to LC–MS analysis.

Quantification was performed using standard curves based on the ratio of the external standard signal to the internal standard signal (*r* ^2^ > 0.99). The standard coverage for quantifying each AVA, AVE, and AVC is provided in Supporting Information Tables S2 and S3.

### Statistical Analysis

Statistical evaluation of the significance of the differences between two groups was performed using Student’s *t* test. For experiments comparing multiple groups, the differences were analyzed by carrying out a one-way analysis of variance (ANOVA) and Duncan’s post hoc test using SPSS (version 21). Data were presented as the mean ± SE for the indicated number of independently performed experiments, and *p*-values of <0.05 were considered statistically significant. All data were subjected to outlier evaluation prior to final analysis.

Correlation analysis was conducted using GraphPad Prism (version 10, GraphPad Software, San Diego, CA, USA) to evaluate the relationships between colon pro-inflammatory markers and oat phytochemical metabolites in feces. Data from the DSS group, DSS + OAT group, DSS + LG-OAT group, and DSS + HG-OAT group were analyzed together. Spearman’s rank correlation coefficient was used, and a two-tailed test was applied. A correlation matrix was generated to visualize the relationships between variables, and statistical significance was set at *p* < 0.05. Additionally, scatter plots with linear regression lines were constructed to illustrate significant correlations. A heatmap was generated to present the results based on the correlation coefficient (*r*) values.

## Results and Discussion

### Germination Enhances the Anti-Inflammatory Effects of Oat Seeds in Cells

When macrophages ingest pathogens, they release toxic substances like superoxide anion (O_2_^–^), hydrogen peroxide (H_2_O_2_), and NO to kill bacteria.^30^ Therefore, the concentration of NO is often measured as an evaluation indicator for macrophage inflammation in anti-inflammatory experiments and is used as the screening assay in this study. To determine whether oat phytochemicals are the active anti-inflammatory components in germinated oats, the germinated oat extract was fractionated into three fractions by using an SPE column. These fractions were further analyzed by LC/MS. As shown in Figure 2A–2C, the oat-specific phytochemicals, AVAs, AVCs, and AVEs, were all present in the 100% methanol fraction. Then, the anti-inflammatory effects of these three fractions on LPS-induced NO production were evaluated in the RAW264.7 macrophages. As shown in Figure 2D, the 100% fraction exhibited strong anti-inflammatory effects in a dose-dependent manner, with 20 and 40 μg/mL reducing NO production by 62.0% and 96.3%, respectively. These findings confirm that the phytochemical-rich fraction possesses significant anti-inflammatory properties.

**Figure 2:**
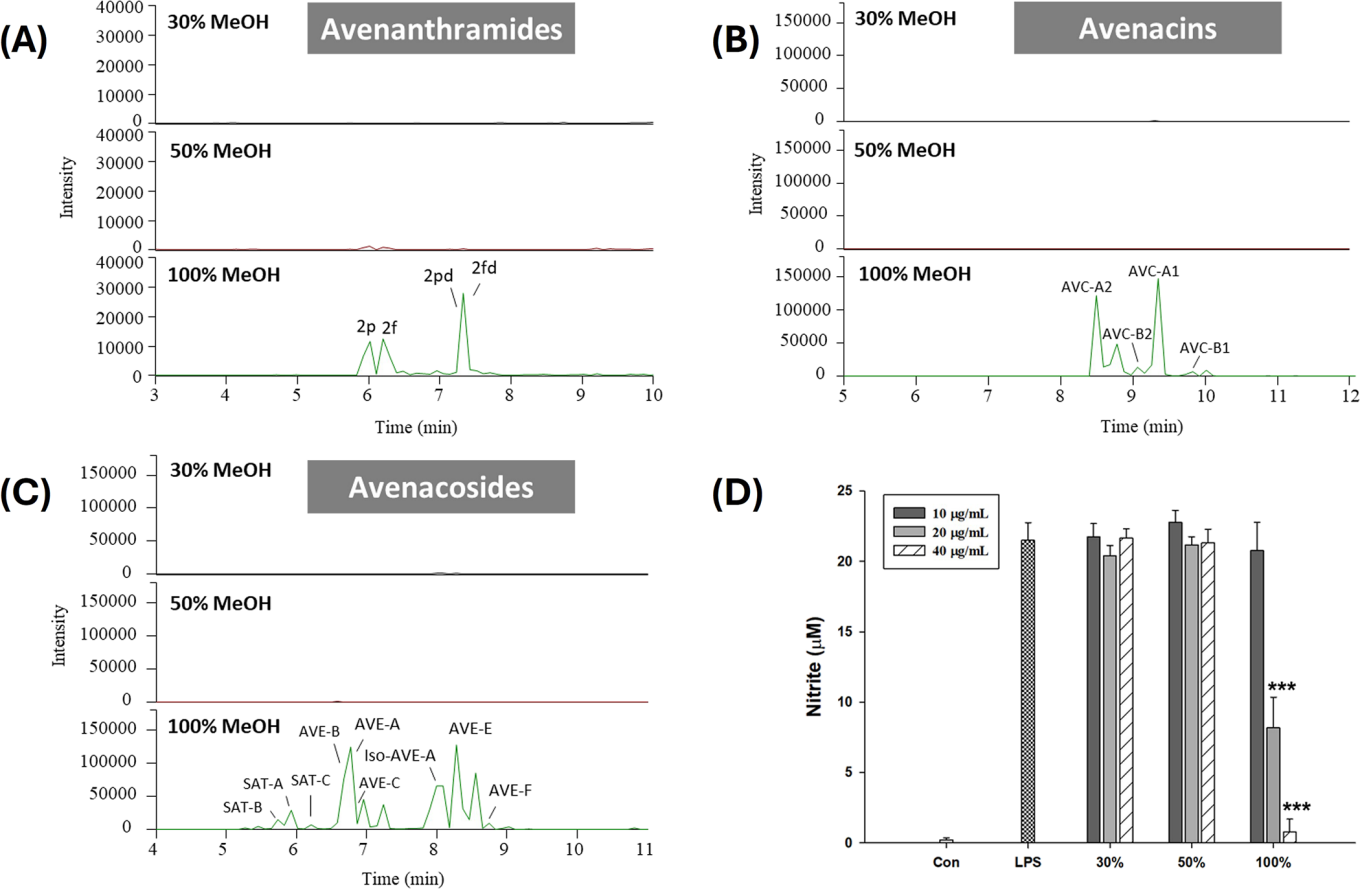
Chemical profiles of avenanthramides (A), avenacins (B), and avenacosides (C) in different fractions of oat extracts from SPE columns analyzed using LC/MS and inhibitory effects of different fractions on LPS-induced NO production in RAW264.7 macrophages (D). Cells were treated with different samples and LPS (100 ng/mL) for 24 h. Samples were dissolved in DMSO. Asterisks indicate a significant difference compared to the LPS group: *** *p* < 0.001.

Using the same NO assay, we compared the anti-inflammatory effects of the phytochemical-rich fraction of 22 different brands of oat seed products before and after germination. As shown in Figure 3, germination enhanced the anti-inflammatory properties of oats, with brands 2, 8, 12, and 15 showing an increase in NO inhibition by 92.7, 63.4, 59.8, and 41.4%, respectively. Compared to germinated oat extract, the raw oat extract showed a weaker anti-inflammatory effect. For example, in the case of brand 2, its nitrite inhibition rate was −7.9%, which was even higher than that of the LPS-induced group. Among these, brand 2 exhibited the strongest effect.

**Figure 3:**
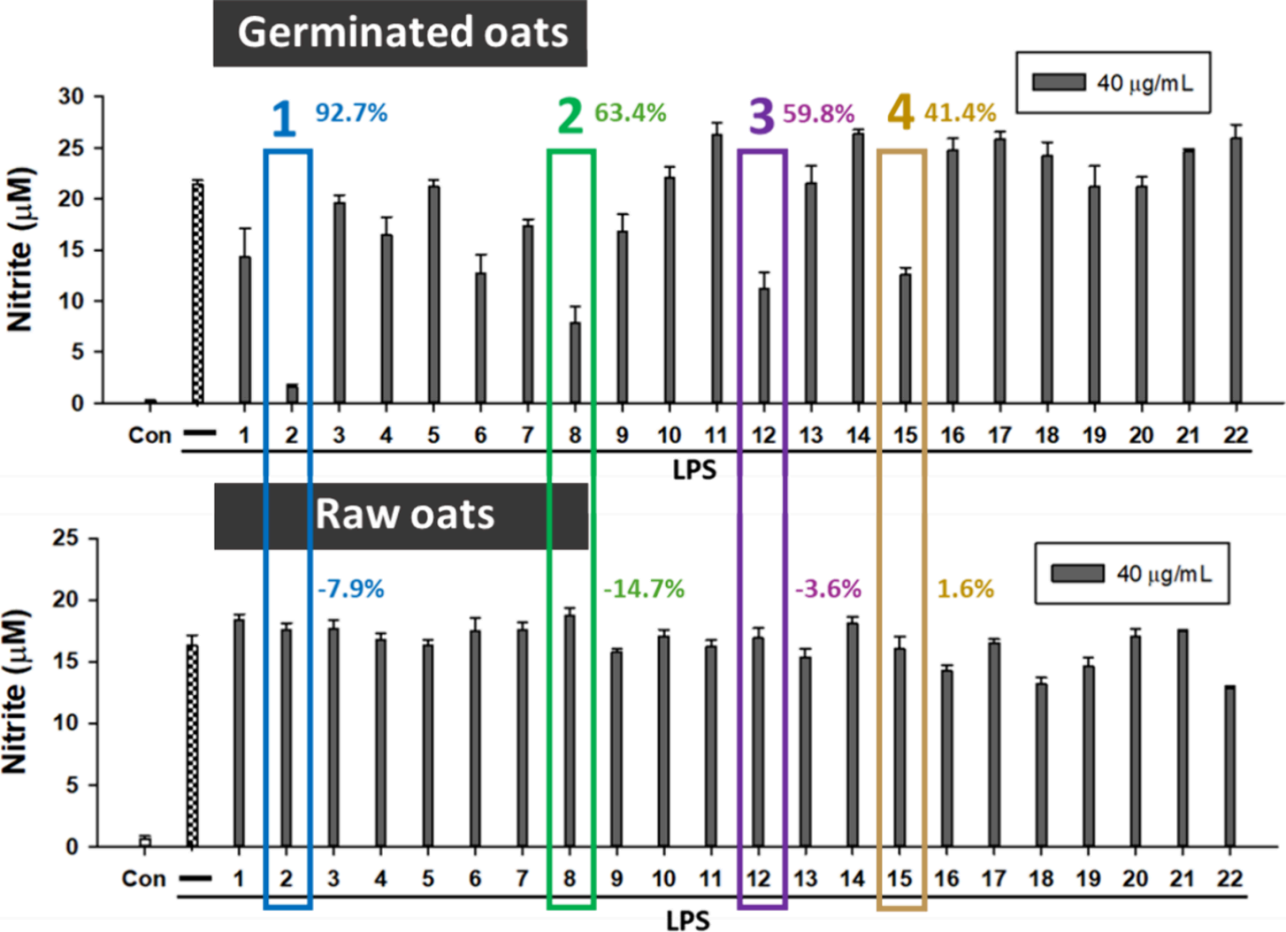
Germination enhances the anti-inflammatory effects of oats in cells. Twenty-two commercially available oat products were germinated, the phytochemical-rich extracts from both raw and germinated oats were prepared using SPE columns. Their anti-inflammatory properties were evaluated at 40 μg/mL using the LPS-induced NO production assay in RAW264.7 macrophages. Samples were dissolved in DMSO. The values are expressed as the mean ± standard deviation (SD).

To confirm whether the anti-inflammatory effects of oat extracts are caused by cytotoxicity, a cell viability test was conducted by using the MTT assay. Figure S1 shows that the cell viability of the germinated oat at the highest concentration (40 μg/mL) did not significantly differ from that of the LPS-induced group, indicating that it does not cause cytotoxicity. In contrast, the cell viability of the raw oat was significantly higher than that of the LPS-induced group. This phenomenon may explain why, in the NO experimental results, the NO content in the raw oat group increased with increasing concentration, possibly related to its ability to promote an increase in cells. The results of cell experiments indicated that the germinated product from brand 2 had a good effect in inhibiting the generation of NO stimulated by LPS. Therefore, we chose the product from brand 2 for subsequent animal experiments.

With respect to the observed variability in anti-inflammatory effects among the germinated oat products (Figure 3), several factors may contribute to these differences. First, genetic variation, both between species (Avena sativa and Avena nuda) and among cultivars within species, can result in differing metabolic responses to germination. Second, agronomic conditions, such as soil quality, climate, and harvest timing, at the growing locations may influence the phytochemical composition and viability of the seeds. Finally, differences in seed quality and intended use may also play a role, as some products are designed for purposes such as forage or ornamental planting rather than optimized for nutritional or health-related applications.

These combined factors likely influence the accumulation of bioactive compounds during germination and may help explain the differences observed in the anti-inflammatory activity across oat products.

### Germination Increases the Levels of Phytochemicals in Oats

Germination led to an overall increase in the content of all AVAs and AVCs, as well as some AVEs (Figure 4 and Table S4). Specifically, for AVAs, the compounds 2c, 2p, 2f, 2cd, 2pd, and 2fd significantly increased by 10.0-, 6.3-, 9.6-, 20.7-, 10.6-, and 4.6-fold, respectively, which is consistent with previous reports.^22^ Furthermore, this study is the first to report an increase in AVCs after germination, with AVC-A2, B2, A1, and B1 contents significantly increasing by 2.5-, 2.2-, 3.6-, and 4.2-fold, respectively. In contrast, although germination resulted in a decrease in certain AVEs, namely, SAT-A, SAT-B, SAT-C, AVE-A, and AVE-B, which significantly decreased by 7.4-, 6.7-, 1.5-, 3.7-, and 3.6-fold, respectively, it significantly increased the levels of AVE-C, Iso-AVE-A, AVE-E, and AVE-F by 1.8-, 3.3-, 3.3-, and 5.0-fold, respectively. Notably, AVE-E has been previously reported to have the strongest anti-inflammatory activity among all of the major AVEs. These fluctuations may be attributed to enzymatic activity induced during germination, which cleaves the attached sugar moieties in major oat AVE compounds, such as AVE-A and AVE-B, into compounds with fewer sugar fragments.^28^ Overall, the total content of these three classes of compounds increased by 2.8-fold, demonstrating that germination is a highly effective strategy for enhancing the accumulation of bioactive compounds, particularly AVAs.

**Figure 4:**
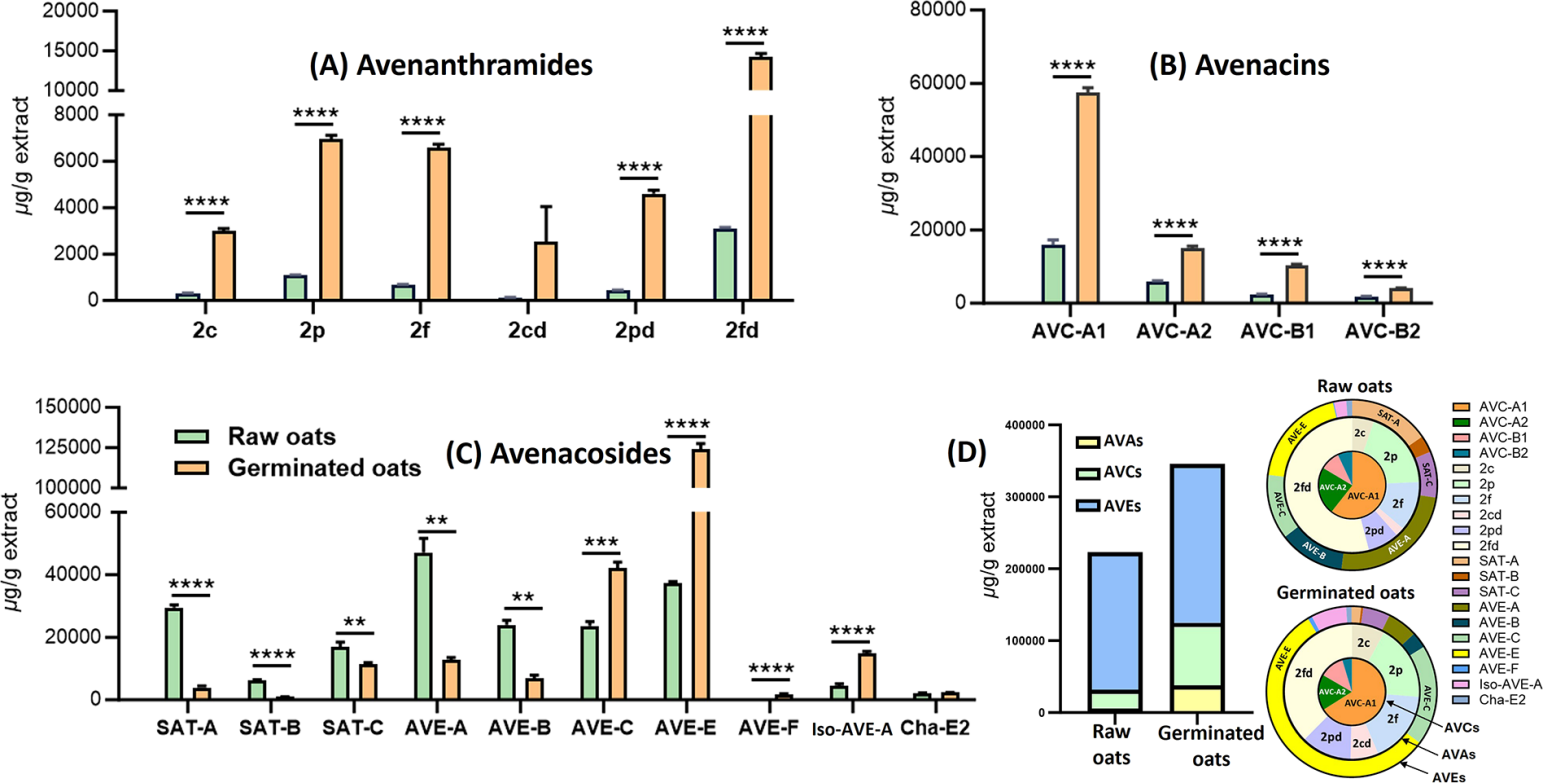
Germination increases the phytochemical content of oats. (A) Avenanthramides; (B) avenacins; (C) avenacosides; data are expressed as means ± SD. The significance of difference was analyzed by *t* test. * *p* < 0.05, ** *p* < 0.01, *** *p* < 0.001, and **** *p* < 0.0001. (D) Total amount and distribution of oat phytochemicals in raw and germinated oats.

### Germination Improves Recovery in DSS-Treated Mice

As shown in Figure 5B and 5C, DSS administration resulted in significant weight loss, confirming the successful establishment of the colitis model. When examining weight change trajectories, both low- and high-dose treatments with the phytochemical-rich extract from germinated oats significantly mitigated weight loss during the seven-day post-DSS period, though no dose-response relationship was observed. After five days of DSS treatment, mice in the DSS group exhibited continuous and significant body weight loss compared to the negative control group (p < 0.005). In contrast, treatment with germinated oat extract promoted body weight recovery, with changes comparable to those of the negative control group. However, the raw oat group did not alleviate the DSS-induced weight loss. During the DSS treatment period, the maximum body weight loss reached 1.6 g in the DSS group and 2.2 g in the raw oat group. In comparison, mice treated with low and high doses of germinated oat extract showed reduced weight loss of only 0.7 g and 1.0 g, respectively, indicating better weight recovery in the germinated oat groups than in the raw oat group.

**Figure 5:**
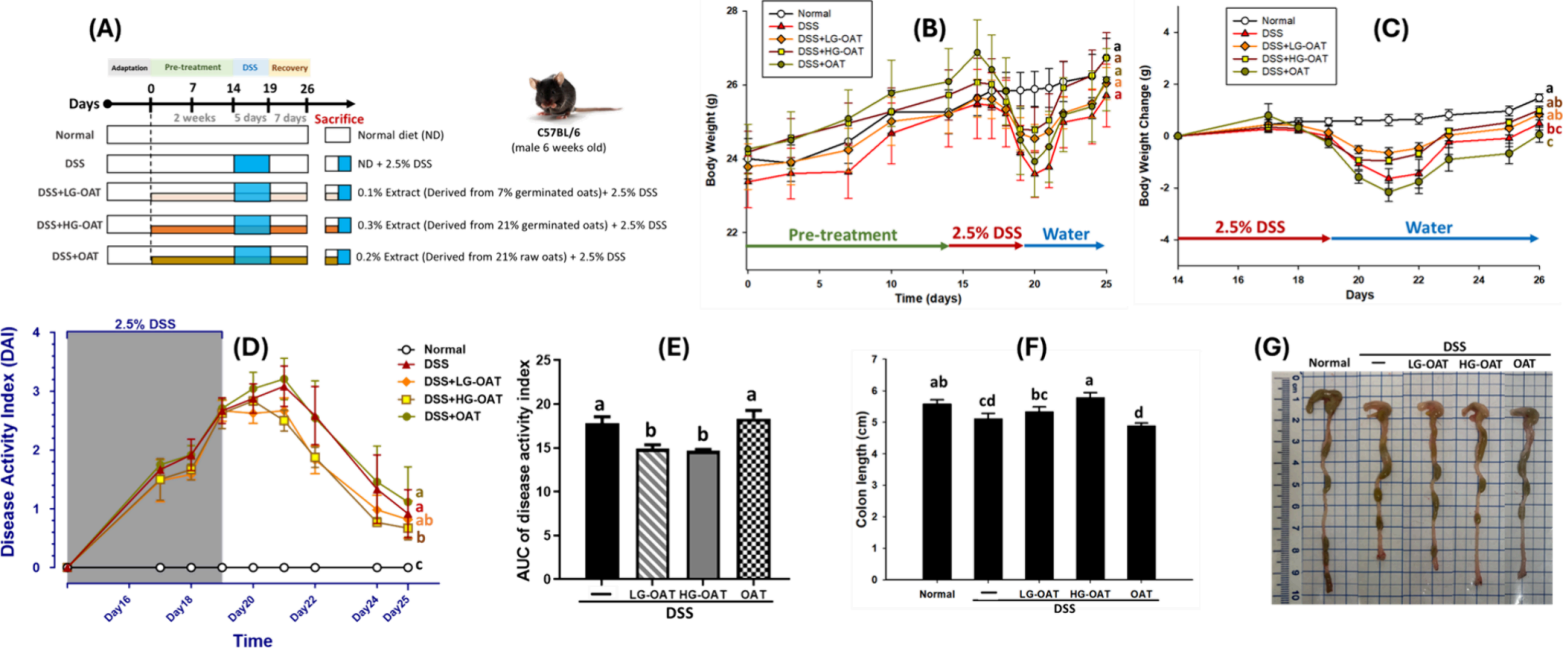
Germination enhances the anti-inflammatory effects of oats on DSS-induced colitis in mice. (A) Experimental design for the *in vivo* study; (B) body weight over the experiment period; (C) body weight change after DSS treatment; (D) disease activity index (DAI) over the experiment period; (E) area under the curve (AUC) of DAI; (F) colon length; (G) representative macroscopic view of the colons. Data are expressed as means ± SE (*n* = 6–8). The significance of difference among the five groups was analyzed by one-way ANOVA and Duncan’s multiple range tests. Different letters indicate significant difference (*p* < 0.05) between groups. LG-OAT: low-dose germinated oats; HG-OAT: high-dose germinated oats; OAT: raw oats.

The changes in the disease activity index (DAI) are presented in Figure 5D,E. The DSS group (1.1 ± 0.14) and the raw oat groups (1.2 ± 0.19) exhibited the highest DAI scores, with no significant difference between them. However, the high-dose germinated oat group (0.7 ± 0.07) significantly attenuated the DSS-induced increase in DAI scores. The area under the curve (AUC) analysis of DAI values during the induction period further supports this observation. Compared to the DSS group (17.8 ± 0.76) and the raw oat group (18.4 ± 0.93), the high- and low-dose germinated oat groups showed reductions of 17.4 and 16.3% in AUC values, respectively, both of which were significantly lower than those of the DSS and raw oat groups. These findings suggest that germinated oats possess superior protective effects against DSS-induced colitis.

In the colitis model, DSS-induced bleeding and inflammation lead to tissue damage, resulting in shortened and thickened colons compared to normal tissue. As shown in Figure 5F and 5G, the colon length of the high-dose germinated oat group (5.8 ± 0.15) was significantly longer than that of the DSS-induced group (5.1 ± 0.17) and was comparable to that of the normal group (5.6 ± 0.13). However, no significant difference was observed between the raw oat group (4.9 ± 0.09) and the DSS group, indicating that while germinated oats effectively alleviated DSS-induced colon shortening, raw oats did not. Additionally, the results show no significant differences in the relative liver weights across groups. However, DSS treatment led to a slight increase in kidney and a significant increase in spleen relative weights (Figure S2). Notably, the high-dose germinated oat and raw oat groups maintained kidney relative weights comparable to those of the normal group, suggesting a potential protective effect.

Overall, these results demonstrate that germinated oats are more effective than raw oats in mitigating DSS-induced colitis symptoms. Supplementary Figure S3 illustrates the food and water intake of mice throughout the experiment. Aside from fluctuations during the five-day DSS induction period due to bleeding and inflammation, no significant differences in food or water consumption were observed among the groups, indicating that the anti-inflammatory effects observed were not influenced by differences in dietary intake.

Although direct studies on the anti-inflammatory effects of germinated oats are limited, our findings are consistent with reports on other germinated or whole grain cereals that show protective effects against intestinal inflammation. Fermented and germinated foxtail millet significantly reduced DSS-induced colitis symptoms and gut microbiota dysbiosis in mice, highlighting the role of processing in enhancing the therapeutic potential of grains.^31^ Ethanol extracts of rice bran and whole grain adlay seeds mitigated colonic damage and inflammation, further supporting the relevance of cereal-derived phytochemicals in modulating colitis.^32^ A whole grain quinoa diet has also been shown to attenuate DSS-induced colitis and reverse gut microbiota imbalance, providing additional evidence that unrefined grains rich in fiber and phytochemicals can promote intestinal health.^33^

### Germination Enhances the Anti-Inflammatory Effects of Oats in Colitis Mice

Pro-inflammatory cytokines contribute to intestinal epithelial damage, immune cell activation, and barrier dysfunction, leading to chronic inflammation and tissue injury. Their strong association with disease activity and severity makes them valuable biomarkers for diagnosing colitis, monitoring disease progression, and assessing treatment efficacy.^34^ The plasma results of (Figure 6A) showed a significant increase in IL-6, IL-1β, and TNF-α after DSS treatment, indicating a higher degree of inflammation. The low-dose germinated oat group significantly reduced IL-6 levels by 52.2%, while the high-dose germinated oat and raw oat groups showed a decreasing trend (27.4 and 17.8%), though not significantly different from the DSS group. In terms of TGF-β expression, both the low- and high-dose germinated oat groups significantly reduced DSS-induced TGF-β levels by 64.1%, whereas the raw oat group achieved a 43.0% reduction. For IL-1β and TNF-α, both germinated oat groups showed a trend toward mitigation, but the differences were not statistically significant compared to the DSS group.

**Figure 6:**
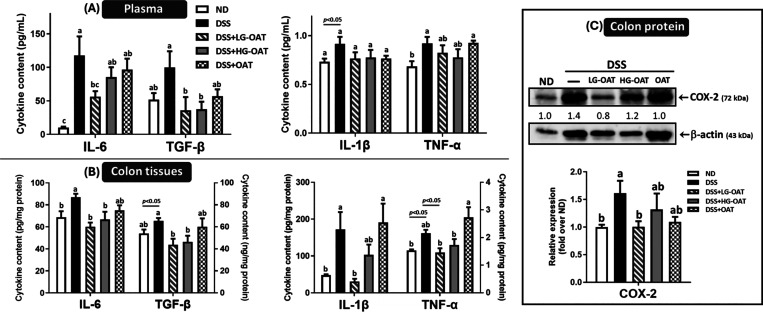
Germination enhances the inhibitory effects of oats on the plasma (A) and colonic tissue (B) levels of pro-inflammatory cytokines and the expression of COX-2 (C) in DSS-induced colitis mice. Pro-inflammatory cytokines were analyzed using commercial ELISA kits and COX-2 protein levels were assessed by Western blot, with β-actin as the loading control. Relative protein levels were normalized to β-actin, and the results are shown as bar graphs. Data are expressed as means ± SE (*n* = 6–8). The significance of difference among the five groups was analyzed by one-way ANOVA and Duncan’s multiple range tests or *t* test. LG-OAT: low-dose germinated oats; HG-OAT: high-dose germinated oats; OAT: raw oats.

Results from colon tissues (Figure 6B) similarly showed a significant increase in the levels of IL-6, TGF-β, IL-1β, and TNF-α after DSS treatment. The low-dose germinated oat group significantly reduced IL-6 by 31.0%, TGF-β by 33.0%, IL-1β by 82.0%, and TNF-α by 32.4%. In contrast, the raw oat group had more modest effects, reducing IL-6 by 13.9% and TGF-β by 8.1%, while actually increasing IL-1β by 10.7% and TNF-α by 26.4%. No significant differences were observed between low- and high-dose germinated oat groups. These findings suggest that germinated oats exhibit stronger anti-inflammatory effects in colon tissues cytokines compared to raw oats, consistent with other indicators of colitis.

The observation of a stronger anti-inflammatory effect in the low-dose germinated oat group compared with the high-dose group is intriguing and warrants further investigation. One possible explanation is the phenomenon of hormesis,^35,36^ where low doses of bioactive compounds can exert beneficial effects, while higher doses may lead to diminished efficacy or even adverse effects. At higher concentrations, certain phytochemicals may activate counterregulatory or stress-related pathways that blunt their anti-inflammatory potential. Further studies involving a broad range of doses would be valuable to define the effective intake range and provide insight into the underlying mechanisms.

Cyclooxygenase-2 (COX-2) is an enzyme that plays a significant role in tissue inflammation by converting arachidonic acid into pro-inflammatory prostaglandins. Increased COX-2 expression in intestinal epithelial cells and macrophages correlates with colitis severity.^37^ Thus, combining COX-2 analysis with pro-inflammatory cytokine levels provides a more comprehensive assessment of inflammation. As shown in Figure 6C, the level of COX-2 expression was significantly elevated in the DSS group compared to that in the ND group. The low-dose germinated oat group significantly reduced COX-2 expression by 37.9%, while the high-dose germinated oat group (18.0%) and the raw oat group (32.3%) showed a decreasing trend but did not significantly differ from the DSS group. Similar patterns were observed between COX-2 expression and cytokine levels, where the low-dose germinated oat group outperformed the high-dose group. This suggests that a dietary-achievable dose, such as 7% in this study, may help maintain gut health. These findings provide insight into optimizing germinated oat dosage for future applications.

Furthermore, a recent review confirmed that whole grain consumption is broadly associated with reduced systemic and intestinal inflammation, partly due to phytochemicals and fiber-mediated gut microbial modulation.^38^ Taken together, our results with germinated oats, which demonstrated increased levels of AVAs, AVCs, and AVEs and improved anti-inflammatory effects, align with findings from related grains. These data support the broader conclusion that grain processing techniques, such as germination or fermentation, can enhance phytochemical content and biological efficacy, thereby reducing inflammation via shared mechanisms across cereal types.

### Oat Phytochemicals Negatively Associate with Colonic Pro-Inflammatory Markers

To investigate the role of oat phytochemicals in the enhanced anti-inflammatory effects observed in phytochemical-rich extracts from germinated oats, we correlated the levels of key oat-derived phytochemicals in mouse feces with colonic pro-inflammatory markers. First, we quantified these phytochemicals in mouse feces using LC/MS. As shown in Figure 7A and Table S5, the germinated oat group exhibited significantly higher levels of AVAs, including 2c and 2f, along with their microbial metabolites DH-2c, DH-2p, DH-2f, and 2cd, 2pd, and 2fd, compared to the raw oats group (Figure 7A). Among these, DH-2f exhibited the greatest difference, with a 101.1-fold increase, followed by 2pd with a 68.9-fold increase and DH-2p with a 26.4-fold increase.

**Figure 7:**
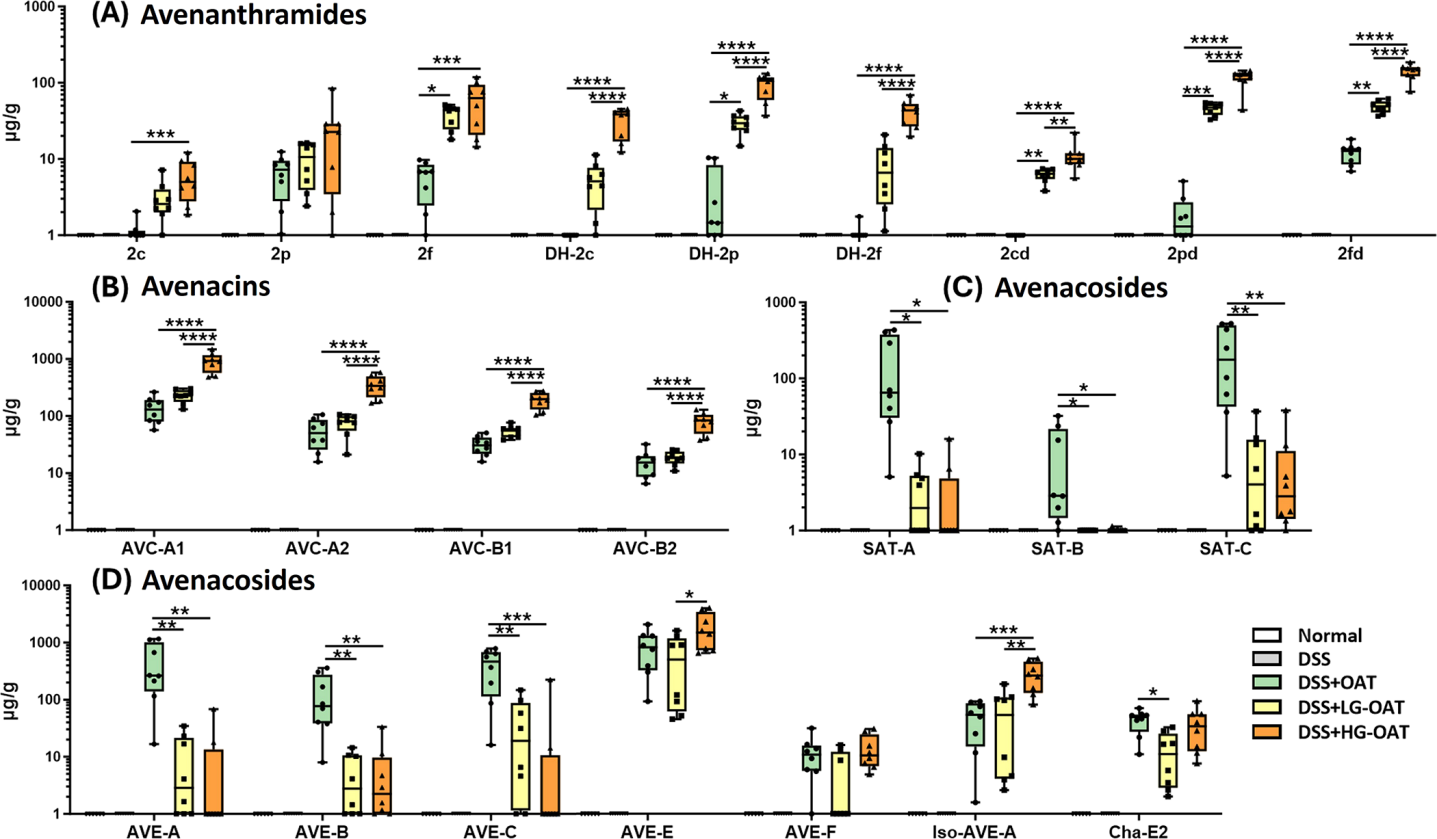
Concentrations of oat phytochemicals and their metabolites in feces from mice treated with low- and high-dose of germinated oats and raw oats. (A) Avenanthramides; (B) avenacins; (C, D) avenacosides. The significance of difference among the raw oat and germinated oat groups was analyzed by one-way ANOVA and Tukey’s multiple range tests. * *p* < 0.05, ** *p* < 0.01, *** *p* < 0.001, and **** *p* < 0.0001. LG-OAT: low-dose germinated oats; HG-OAT: high-dose germinated oats; OAT: raw oats.

Similarly, AVCs, including AVC-A1, AVC-A2, AVC-B1, and AVC-B2, were significantly higher in the feces of the germinated oat group, with increases of 6.54-, 6.23-, 5.85-, and 4.75-fold, respectively (Figure 7B). In contrast, germination led to a significant reduction in major AVEs such as AVE-A, AVE-B, SAT-A, and SAT-B, likely due to hydrolysis into AVEs with fewer sugar moieties such as AVE-E and AVE-F (Figure 4C). As a result, the germinated oats group, particularly the high-dose group, had significantly lower levels of AVE-A (58.4-fold decrease), AVE-B (19.2-fold decrease), SAT-A (59.4-fold decrease), and SAT-B (55.9-fold decrease), while AVE-E and AVE-F increased by 2.1- and 1.2-fold, respectively, compared to the raw oats group (Figure 7C,D).

To assess whether oat phytochemicals contribute to the anti-inflammatory effects of germinated oats, we conducted a correlation analysis comparing colonic tissue levels of key pro-inflammatory cytokines (IL-6, TNF-α, IL-1β, and TGF-β) with the concentrations of 23 oat-derived metabolites detected in feces. As shown in Figure 8A, all AVAs, including C-type AVAs (2c, 2p, 2f), their microbial metabolites (DH-2c, DH-2p, DH-2f), and A-type AVAs (2cd, 2pd, 2fd), exhibited a significant negative correlation with two or more pro-inflammatory cytokines, indicating that AVAs play a critical role in the anti-inflammatory effects observed in the colitis mouse model. Additionally, AVC-A1 and AVC-B1 showed a significant negative correlation with both TNF-α and TGF-β, AVC-B2 correlated negatively with TGF-β, AVE-E correlated negatively with IL-6, and Iso-AVE-A correlated negatively with TNF-α.

**Figure 8:**
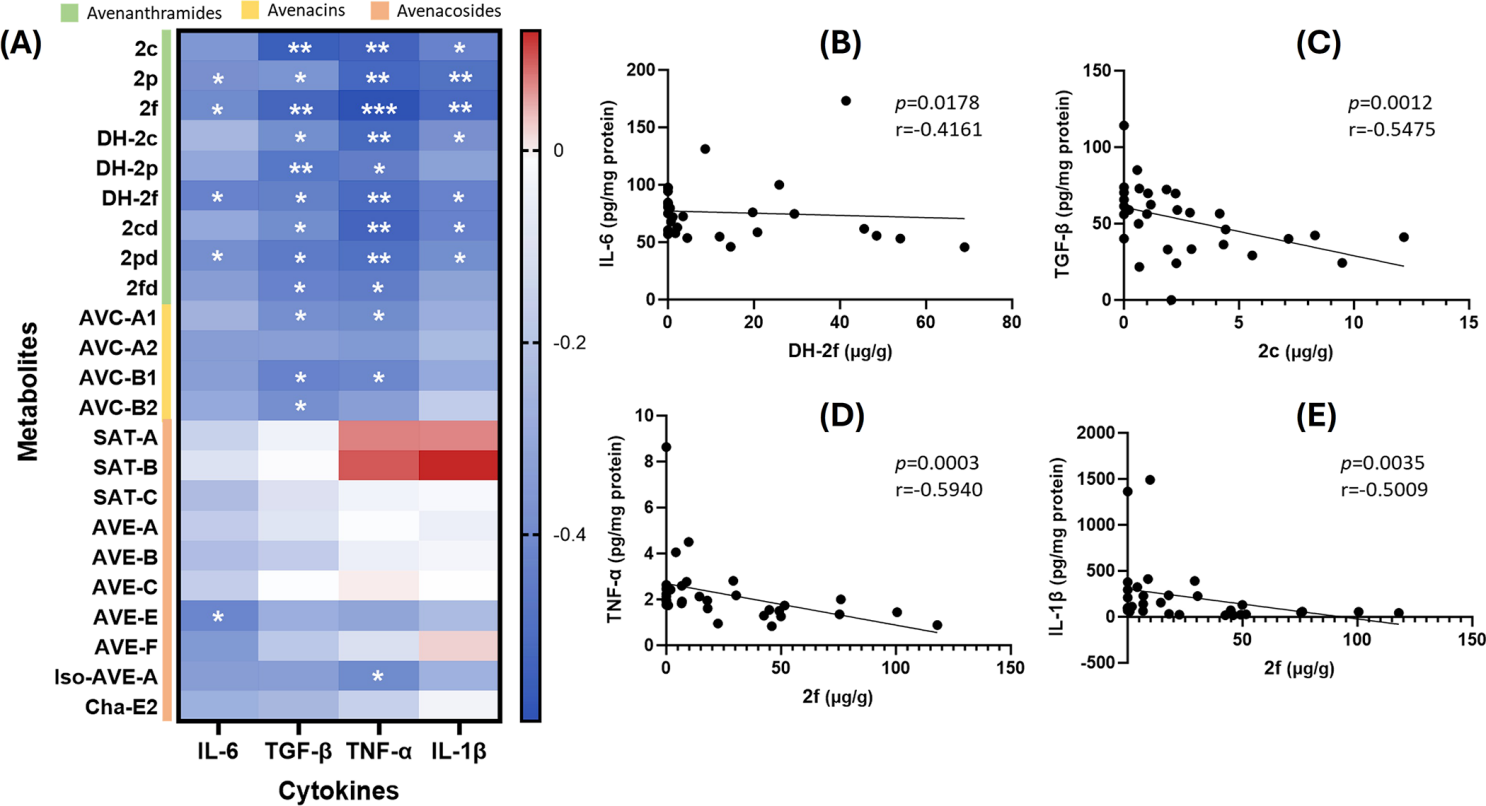
Oat phytochemicals negatively correlate with colonic pro-inflammatory markers. (A) Heatmap showing the correlation between 23 oat phytochemicals and metabolites and four pro-inflammatory markers (IL-6, TGF-β, TNF-α, and IL-1β); (B) correlation of IL-6 with DH-2f; (C) correlation of TGF-β with 2c; (D) correlation of TNF-α with 2f and (E) correlation of IL-1β with 2f. Color intensity indicates the degree of correlation (blue represents a negative correlation, red shows a positive correlation). * *p* < 0.05, ** *p* < 0.01 and *** *p* < 0.001.

Figure 8B–E highlights the metabolites most significantly correlated with the four cytokines, particularly DH-2f, 2c, and 2f. Among these, 2f demonstrated the highest potential as an anti-inflammatory metabolite. These findings align with previous research published in 2020,^10^ which compared the effects of different oat AVAs on nitrite levels in LPS-induced RAW264.7 macrophages and identified 2f as having the strongest anti-inflammatory effect. In the correlation analysis for IL-6, the microbial metabolite DH-2f (*p* = 0.0178, *r* = −0.4161) exhibited a stronger negative correlation than 2f (*p* = 0.0289, *r* = −0.386). Similarly, for TGF-β, DH-2p (*p* = 0.0079, *r* = −0.461) showed a more significant negative correlation than that of 2p (*p* = 0.0459, *r* = −0.355). These results suggest that gut microbiota composition may influence the anti-inflammatory effects of oat phytochemicals in colitis.

Moreover, it is possible that AVAs, AVEs, and AVCs act synergistically to enhance the overall anti-inflammatory efficacy, potentially by targeting different inflammatory pathways or modulating each other’s bioavailability and activity. Further investigation into the synergistic interactions among these compounds is warranted.

In this study, we inferred microbial involvement based on the presence of microbial-derived metabolites, such as DH-2p and DH-2f,^20^ of oat phytochemicals in fecal samples, and their significant correlations with inflammatory markers. However, we acknowledge that the interaction between the diet and gut microbiota is highly complex and dynamic and cannot be fully explained by metabolite profiling alone. Comprehensive microbiome analysis, such as 16S rRNA sequencing or metagenomic approaches, would be necessary to clarify the compositional and functional shifts in microbial communities in response to germinated oat intake.

Incorporating germinated oat extracts into commercial formulations appears feasible, particularly in forms such as beverages, bars, or supplements. However, the stability of these bioactive compounds during food processing and storage is a critical factor to consider. While some studies have shown that avenanthramides and related compounds are relatively stable under moderate thermal conditions,^39,40^ further investigation is needed to evaluate their retention in various processing environments.

In summary, germination enhances the anti-inflammatory properties of oats in both cells and DSS-induced colitis in mice by increasing the levels of bioactive phytochemicals. Correlation analysis showed a significant inverse relationship between pro-inflammatory cytokines and phytochemical content in feces, especially AVAs and their microbial metabolites. These results suggest that specific oat phytochemicals are key contributors to the anti-inflammatory effects and that gut microbiota may modulate their activity. Notably, germination improved the anti-inflammatory effects in only certain oat varieties, indicating that the seed type plays a role and warrants further investigation into the mechanisms involved.

## Supplementary Material



## Notes

Characteristics of the 22 oat products used in the study, standard curves and quantification coverage of AVAs, AVCs, and AVEs, concentrations of phytochemicals in extracts (μg/g extract) from raw and germinated oat seeds of product 2, concentrations of oat phytochemicals and metabolites in feces (μg/g feces) of mice treated with germinated oats and raw oats, effect of oat extracts on cell viability, relative weights of the liver, kidneys, and spleen in mice, and the food and water intake in the animal experiment (PDF)
