# Supplementary material for: Phytochemical-Rich Germinated Oats as a Novel Functional Food To Attenuate Gut Inflammation
Source: J Agric Food Chem. 2025 Jun 12;73(25):15706–16. doi: 10.1021/acs.jafc.5c02993 (PMC12203580; doi:10.1021/acs.jafc.5c02993)
Supplement: Supplementary file 1 [file jf5c02993_si_001.pdf]

Supporting Information

Phytochemical-rich germinated oats as a novel functional food to attenuate gut inflammation

Pei-Sheng Lee[1,#], Juanjuan Hu[1,#], and Shengmin Sang[1,2,*]

[1]Laboratory for Functional Foods and Human Health, Center for Excellence in Post-Harvest Technologies, North Carolina Agricultural and Technical State University, Kannapolis, North Carolina 28081, United States

[2]Center for Gastrointestinal Biology and Disease, University of North Carolina, Chapel Hill, North Carolina 27599, United States

[#]These authors have contributed equally to this work.

*Please send all correspondence to:

Dr. Shengmin Sang

E-mail: ssang@ncat.edu

Table S1. Characteristics of 22 commercial oat seed products

Brand	Growing Region	Oat Species	Growth Characteristics	Hull Presence	Uses
1	Iowa, USA	*Avena sativa* L.	Fall planting, blooms in fall	Hulled	Forage for wildlife
2	Southern Iowa, USA	*Avena sativa* L.	Spring planting, blooms in spring and winter	Hulled	Planting
3	Iowa, USA	Data not available	Adaptable for year-round planting	Hulled	Cat Grass Seeds
4	Willamette Valley, Oregon, USA	*Avena sativa* L.	Adaptable for year-round planting	Hulled	Planting
5	Florida, USA	*Avena sativa* L.	Adaptable for year-round planting	Hulled	Cat Grass Seeds
6	South Dakota, USA	Data not available	Adaptable for year-round planting	Hulled	Cat Grass Seeds
7	Wisconsin, USA	*Avena sativa* L.	Late summer to fall planting, blooms in summer	Hulled	Livestock forage
8	American Midwest	*Avena sativa* L.	Adaptable for year-round planting	Hulled	Cat Grass Seeds
9	Montana, USA	*Avena nuda*	Early spring planting, harvested in late summer	Hull-less	Planting
10	USA	*Avena sativa* L.	Data not available	Hulled	Human consumption and livestock feed
11	USA	*Avena sativa* L.	Adaptable for year-round planting	Hulled	Cat Grass Seeds
12	Lehi, Utah, USA	*Avena sativa* L.	Data not available	Hulled	Planting
13	USA	*Avena sativa* L.	Late summer or early fall planting	Hulled	Planting
14	Michigan, USA	Data not available	Adaptable for year-round planting	Hulled	Cat Grass Seeds
15	Iowa	*Avena sativa* L.	Data not available	Hulled	Planting
16	Salt Lake, Utah, USA	*Avena nuda*	Spring planting	Hulled	Planting
17	Salt Lake, Utah, USA	*Avena nuda*	Spring planting	Hull-less	Planting
18	Salt Lake, Utah, USA	*Avena nuda*	Data not available	Hull-less	Human consumption
19	USA	*Avena sativa* L.	Data not available	Hulled	Deer attraction and feeding
20	Salt Lake, Utah, USA	*Avena sativa* L.	Data not available	Hulled	Planting and human consumption
21	Salt Lake, Utah, USA	Data not available	Adaptable for year-round planting	Hulled	Planting
22	Salt Lake, Utah, USA	*Avena sativa* L.	Spring planting	Hull-less	Planting

Table S2. Standard curves and quantification coverage of avananthramides (2c, 2p, 2f, 2cd, 2pd, and 2fd), avenacins (AVC-A1, -A2, -B1, and -B2) and avenacosides (AVE-A, -B, -C, -E, and -F, Iso-AVE-A, SAT-A, -B, and -C, and Cha-E_2) in oat extracts using authentic standards

Analytes	Standard curves	R^2	Coverage
2c	$y = 0.0014x - 0.0014$	0.9970	2c
2p	$y = 0.0189x - 0.0044$	0.9991	2p
2f	$y = 0.0412x - 0.0159$	0.9988	2f
2cd	$y = 0.007x - 0.0028$	0.9993	2cd
2pd	$y = 0.0399x - 0.000008$	0.9995	2pd
2fd	$y = 0.0393x - 0.0102$	0.9994	2fd
AVE-A	$y = 0.3236x + 0.598$	0.9952	AVE-A, Iso-AVE-A
AVE-B	$y = 0.166x + 0.1024$	0.9929	AVE-B, AVC-A1, AVC-A2, AVC-B1, AVC-B2
AVE-C	$y = 0.0157x + 0.0314$	0.9924	AVE-C, AVE-F
AVE-E	$y = 0.1217x + 0.1532$	0.9977	AVE-E
SAT-A	$y = 0.1357x + 0.0381$	0.9969	SAT-A
SAT-B	$y = 0.1895x + 0.1255$	0.9942	SAT-B
SAT-C	$y = 0.0495x + 0.021$	0.9971	SAT-C, Cha-E_2

Table S3. Standard curves and quantification coverage of avananthramides (2c, 2p, 2f, 2cd, 2pd, 2fd, DH-2c, DH-2p, and DH-2f), avenacins (AVC-A1, -A2, -B1, and -B2) and avenacosides (AVE-A, -B, -C, -E, and -F, Iso-AVE-A, SAT-A, -B, and -C, and Cha-E_2) in fecal samples using authentic standards

Analytes	Standard curves	R^2	Quantified compounds
2c	$y = 0.0004x + 0.00004$	0.9957	2c
2p	$y = 0.0058x + 0.0043$	0.9983	2p
2f	$y = 0.0106x + 0.0098$	0.9982	2f
2cd	$y = 0.0024x + 0.0005$	0.9998	2cd
2pd	$y = 0.0123x + 0.0122$	0.9993	2pd
2fd	$y = 0.0105x + 0.0097$	0.9998	2fd
DH-2c	$y = 0.0112x + 0.0024$	0.9995	DH-2c
DH-2p	$y = 0.0036x + 0.0020$	0.9986	DH-2p
DH-2f	$y = 0.0076x + 0.0023$	0.9985	DH-2f
AVE-A	$y = 0.1329x + 0.0384$	0.9996	AVE-A, Iso-AVE-A
AVE-B	$y = 0.0617x + 0.0002$	0.9969	AVE-B, AVC-A1, AVC-A2, AVC-B1, AVC-B2
AVE-C	$y = 0.0062x + 0.0008$	0.9984	AVE-C, AVE-F
AVE-E	$y = 0.0371x - 0.0204$	0.9903	AVE-E
SAT-A	$y = 0.0487x - 0.0004$	0.9969	SAT-A
SAT-B	$y = 0.1065x + 0.0009$	0.9975	SAT-B
SAT-C	$y = 0.0208x - 0.0004$	0.9998	SAT-C, Cha-E_2

Table S4. Concentrations of phytochemicals in extracts (μg/g extract) from raw and germinated oat seeds of product 2

		Raw oats	Germinated oats
Avenanthramides	2c	303.1±11.8	3017.1±97.6
	2p	1095.5±19.6	6954.1±170.4
	2f	688.5±6.7	6598.5±141.1
	2cd	122.9±5.2	2542.0±1513.2
	2pd	436.8±6.2	4604.7±154.9
	2fd	3110.0±51.0	14261.0±411.4
Avenacins	AVC-A1	15863.4±1391.8	57466.3±1369.2
	AVC-A2	5974.9±154.0	15075.8±483.5
	AVC-B1	2462.0±36.6	10420.6±232.7
	AVC-B2	1846.7±22.0	4048.2±122.4
Avenacosides	SAT-A	29452.0±882.5	3955.5±472.8
	SAT-B	6147.5±241.4	923.9±49.8
	SAT-C	16785.4±1635.0	11359.7±502.0
	AVE-A	47148.0±4537.0	12767.0±785.4
	AVE-B	23937.6±1527.4	6742.1±1122.1
	AVE-C	23630.0±1326.0	42145.0±1816.0
	AVE-E	37130.9±623.8	124138.0±3444.0
	AVE-F	366.0±20.1	1826.0±107.7
	Iso-AVE-A	4513.2±657.2	14903.0±578.8
	Cha-E2	2146.0±54.0	2253.0±85.5

Table S5. Concentrations of oat phytochemicals and metabolites in feces (μg/g feces) of mice treated with germinated oats and raw oats

		Normal	DSS	DSS+OAT	DSS+LG-OAT	DSS+HG-OAT
Avenanthramides	2c	0.0±0.0	0.0±0.0	0.9±0.6	3.1±1.9	6.0±3.6
	2p	0.0±0.0	0.0±0.0	6.7±3.9	10.0±6.0	24.6±26.6
	2f	0.0±0.0	0.0±0.0	5.7±3.2	38.2±12.7	60.2±38.6
	DH-2c	0.0±0.0	0.0±0.0	0.0±0.0	5.3±3.4	32.0±13.5
	DH-2p	0.0±0.0	0.0±0.0	3.5±4.2	29.7±8.7	93.5±33.9
	DH-2f	0.0±0.0	0.0±0.0	0.4±0.6	8.4±6.9	41.7±16.2
	2cd	0.0±0.0	0.0±0.0	0.0±0.0	6.3±1.2	11.2±5.0
	2pd	0.0±0.0	0.0±0.0	1.7±1.7	46.5±8.4	117.2±32.2
	2fd	0.0±0.0	0.0±0.0	12.1±3.7	49.6±8.9	143.0±33.4
Avenacins	AVC-A1	0.0±0.0	0.0±0.0	138.7±70.5	227.3±59.4	915.4±339.0
	AVC-A2	0.0±0.0	0.0±0.0	55.5±32.6	76.6±29.1	353.8±146.3
	AVC-B1	0.0±0.0	0.0±0.0	31.6±11.8	54.2±13.0	192.4±61.8
	AVC-B2	0.0±0.0	0.0±0.0	15.7±8.2	18.8±5.1	80.4±31.2
Avenacosides	SAT-A	0.0±0.0	0.0±0.0	166.8±180.0	3.1±3.7	2.8±5.8
	SAT-B	0.0±0.0	0.0±0.0	10.1±12.3	0.1±0.2	0.2±0.4
	SAT-C	0.0±0.0	0.0±0.0	243.2±223.3	9.5±12.7	8.2±12.7
	AVE-A	0.0±0.0	0.0±0.0	479.3±452.1	10.1±13.1	10.8±24.1
	AVE-B	0.0±0.0	0.0±0.0	134.7±132.4	5.2±5.7	7.0±11.2
	AVE-C	0.0±0.0	0.0±0.0	419.5±294.9	43.4±54.6	30.0±79.1
	AVE-E	0.0±0.0	0.0±0.0	876.7±643.7	612.1±613.4	1868.2±1319.1
	AVE-F	0.0±0.0	0.0±0.0	11.8±9.4	4.7±6.8	14.2±9.7
	Iso-AVE-A	0.0±0.0	0.0±0.0	50.4±34.8	64.3±68.8	276.7±161.4
	Cha-E2	0.0±0.0	0.0±0.0	44.0±19.0	13.5±12.0	38.1±29.0

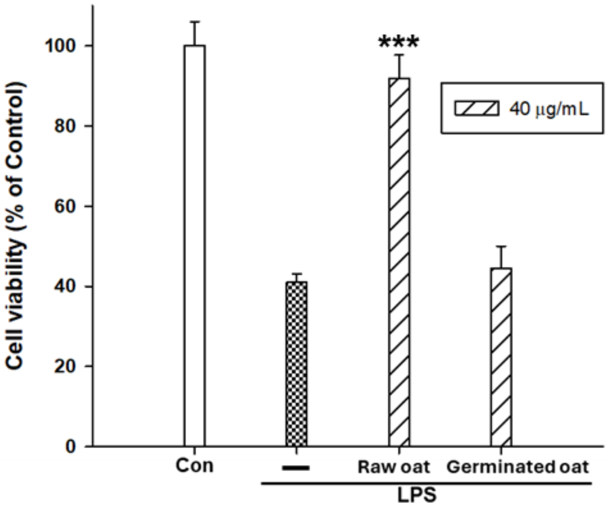

Figure S1. Cell viability in RAW 264.7 macrophage. Cells were treated either without LPS (negative control) or with LPS (100 ng/mL) in the absence or presence of 40 μg/mL raw oat extract or germinated oat extract for 24 hours. Cell viability was assessed using the MTT assay. Extracts were dissolved in DMSO. Data are expressed as the mean ± standard deviation (SD). An asterisk means a significant difference compared to the LPS group: (*) $p < 0.05$, (**) $p < 0.01$, and (***) $p < 0.001$.

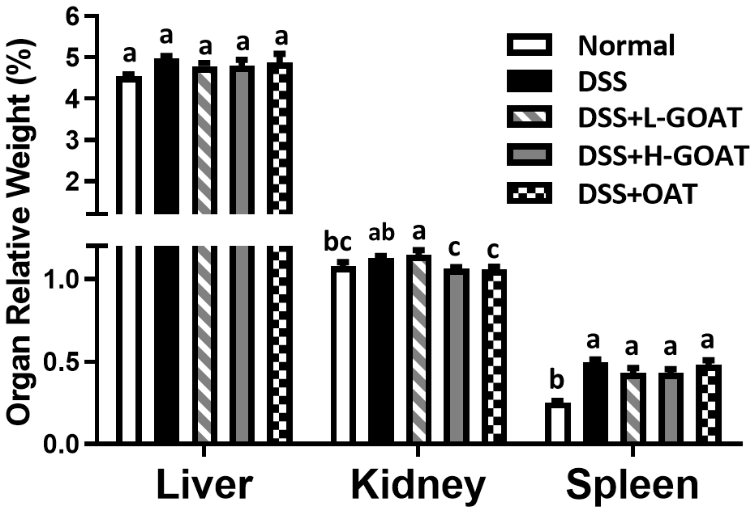

Figure S2. Effect of germinated oats and raw oats on organ relative weight in DSS-induced colitis in mice. Data are expressed as means ± SE. The significance of difference among the five groups was analyzed using one-way ANOVA and followed by Duncan's multiple range test.

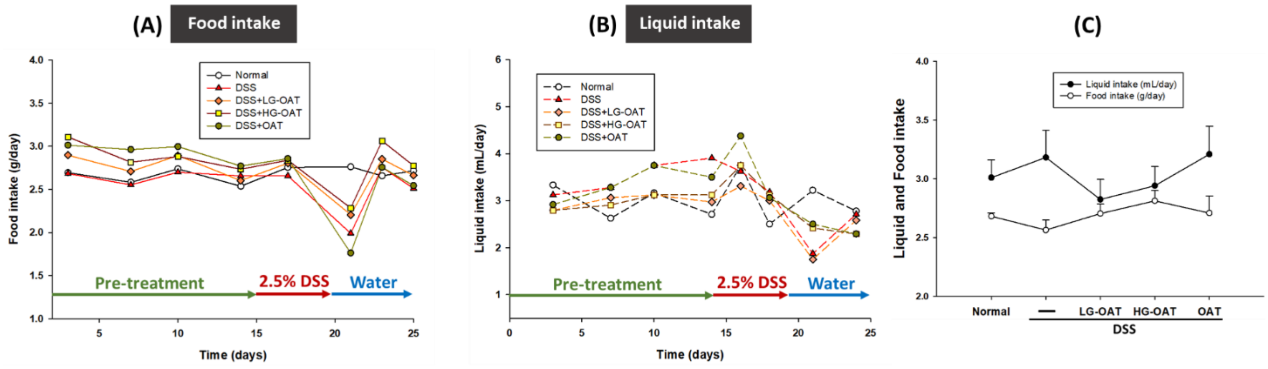

Figure S3. Food and liquid intake during the experiment period in DSS-induced colitis in mice. (A) Daily food intake; (B) daily liquid intake; (C) average food and liquid intake over the entire study period.
